# A multicentre cross-sectional survey of the core competence of paediatric emergency nurses

**DOI:** 10.3389/fped.2025.1635942

**Published:** 2025-07-24

**Authors:** Yi Tao, Wenjing Li, Li Zhang, Shuang Zhan, Man Wang, Mei Feng

**Affiliations:** ^1^ Department of Emergency, Children’s Hospital of Chongqing Medical University, Chongqing, China; ^2^ National Clinical Research Center for Child Health and Disorders, Ministry of Education Key Laboratory of Child Development and Disorders, Chongqing Key Laboratory of Child Rare Diseases in Infection and Immunity, Chongqing, China

**Keywords:** pediatric, emergency, nurse, core competencies, cross-sectional survey

## Abstract

**Introduction:**

To understand the core competency levels of paediatric emergency nurses and factors that influence these levels, and to provide a basis for improving the core competencies of paediatric emergency nurses via education and training, and to provide a reference for managers for implementing scientific management models.

**Methods:**

From October to November 2022, convenience sampling was used to select 540 paediatric emergency nurses from 9 provinces and cities across the country as survey subjects. A self-designed “Self-evaluation questionnaire on core competencies of paediatric emergency nurses” was used for survey analysis.

**Results:**

The average score of the core competency items among paediatric emergency nurses was 4.06 ± 0.74, the highest score was 4.51 ± 0.62 in the professional attitude dimension, and the lowest score was 3.70 ± 0.81 in the professional development ability dimension. The results of the multiple regression analysis revealed that the hospital level and emergency management experience are the factors that influence the core competencies of paediatric emergency nurses. The core competencies of paediatric emergency nurses are at an average level and are influenced by various factors.

**Discussion:**

Paediatric emergency nursing managers should develop comprehensive, systematic, and professional training plans, implement scientific management models in paediatric emergency management work, and increase the core competency levels of paediatric emergency nurses.

## Introduction

The National Nursing Career Development Plan (2021–2025) proposes to strengthen the core competencies of nurses in emergency care and paediatric nursing via training to improve the level of specialized nursing skills. Paediatric emergency nurses are the first point of contact for children with acute and critical illnesses, and their service targets are children aged ≤18 years with such conditions. The types of acute and critical illnesses vary in each age group, and there is a wide range of illnesses and critical conditions. Infants and young children cannot accurately describe their illnesses, which leads to a lack of accuracy in the collection of complaints and illnesses. This limitation poses a great challenge to paediatric emergency nursing care. The core competencies of nursing staff are their professional knowledge, skills, attitudes, and personal attributes. Paediatric emergency nurses face great challenges due to changes in fertility policies and the development of high-quality nursing care. Their core competencies directly affect the care outcomes for children in emergency care. There are explicit and implicit factors in core competencies, and implicit factors are difficult to determine directly through external assessment.

To better understand the core competencies of paediatric emergency nurses, this group first examined the improvement in the core competencies of emergency nurses and paediatric nurses domestically and internationally and the related theoretical basis through a literature research method. The dimensions and initial entry pool for the Core Competency Self-Assessment Questionnaire for Paediatric Emergency Nurses was prepared through a theoretical analysis and group discussion. Through descriptive qualitative research methods, interviews were subsequently conducted with paediatric emergency nurses to supplement the dimensions and initial pool of items to form the first draft of the Paediatric Emergency Nurses’ Core Competency Self-Assessment Questionnaire. Finally, using the Delphi Expert Correspondence Method, the 20 experts who were consulted via correspondence all came from the emergency departments of the top 10 children's specialty hospitals in China, according to the Fudan University Hospital Ranking. The familiarity of the experts was 0.800, the judgment was coefficient 0.918, and the authority coefficient was 0.859. The questionnaire was revised according to expert feedback, and the final draft was formed. The questionnaire was tested for reliability and validity using the questionnaire survey method and further revised to form the formal self-assessment questionnaire. It evaluates core competencies across four primary dimensions (professional attitude, clinical expertise, comprehensive ability, and professional development capacity) containing 31 validated items. The comprehensive ability dimension encompasses three key components: communication and coordination proficiency, healthcare management competency, and clinical critical thinking capacity. This study conducted a multicentre cross-sectional survey and data analysis based on the formal Paediatric Emergency Nurses’ Core Competency Self-Assessment Questionnaire. The aim of this study is to understand the levels of paediatric emergency nurses’ core competencies and the factors affecting them. This understanding will provide the basis for education and training to improve the core competencies of paediatric emergency nurses and provide a reference for the implementation of a scientific management model by management staff.

## Participants and methods

### Participants

Based on the professional requirements for paediatric emergency nursing roles, participants were selected from the emergency departments of national paediatric specialty hospitals, maternal and child health hospital emergency departments, and paediatric emergency outpatient units in general hospitals. The inclusion criteria were as follows: (1) associate degree or higher education; (2) registered nurse status; (3) ≥1 year of paediatric emergency nursing experience; and (3) voluntary participation. The exclusion criteria were as follows: (1) Individuals currently not engaged in paediatric emergency nursing practice.

### Research instruments

The study employed a self-developed Paediatric Emergency Nurse Core Competency Self-Assessment Questionnaire as the research instrument. The validation results demonstrated excellent psychometric properties, with a Cronbach's *α* coefficient of 0.881 and a content validity index (CVI) of 0.889. The questionnaire comprises three sections: (1) Instructions: detailed guidance for standardized completion; (2) demographic profile: age, educational background, professional title, marital status, employment type, years of paediatric emergency experience, hospital classification, and participation in Paediatric Advanced Life Support (PALS) or Paediatric Basic Life Support (PBLS) training programs; and (3) core assessment module: featuring a 5-point Likert scale (1 = strongly disagree; 2 = disagree; 3 = neutral; 4 = agree; 5 = strongly agree), with higher composite scores indicating superior core competency levels.

### Research methodology

A questionnaire-based survey was conducted using convenience sampling from October–November 2022. We employed a stratified sampling strategy to obtain a nationally representative sample of pediatric emergency nurses, systematically ensuring geographical coverage across China's seven major regions while incorporating both tertiary and secondary healthcare institutions with pediatric emergency units. The sampling framework deliberately balanced nurse experience levels (1–5 years and ≥5 years) and professional ranks (Staff Nurses, Nurse Practitioners, and Senior Nurses), with each tier representing no less than 20% of the sample. This hybrid approach combining stratified targeting with convenience sampling provided operational advantages for multicenter implementation, including feasibility for rapid large-scale data collection and efficient resource utilization within constrained timelines, while acknowledging inherent limitations such as potential selection bias and possible underrepresentation of certain subpopulations despite the stratification efforts. Prior to data collection, researchers coordinated online with unit nurse managers to provide standardized training on survey administration. Uniform instructional protocols were implemented to explain the study's purpose and significance. The unit managers subsequently conducted centralized staff training and distributed the questionnaire via the Wenjuanxing platform (a China-based equivalent to Qualtrics®), ensuring compliance with data security protocols (HTTPS encryption, IP restrictions).

### Statistical analysis

Data analysis was performed using SPSS 26.0. Univariate comparisons of core competency scores across demographic variables were conducted through one-way ANOVA and independent t-tests. Variables demonstrating statistical significance (*P* < 0.05) in univariate analyses were entered into a multiple linear regression model to identify independent predictors of paediatric emergency nurses’ core competencies. The regression analysis adhered to standard diagnostic procedures, including collinearity assessment (VIF <5) and residual normality verification.

## Results

### Participant characteristics

A total of 540 questionnaires were distributed, with 538 valid responses received (response rate: 99.6%). Among them, Chongqing Municipality (210 cases), Sichuan Province (72 cases), Beijing (58 cases), Yunnan Province (49 cases), Jiangxi Province (40 cases), Xinjiang Uygur Autonomous Region (30 cases), Hunan Province (27 cases), Shaanxi Province (26 cases), and Guangdong Province (26 cases).The detailed demographic characteristics of the participants are presented in Table 1.

**Table 1:** General information of the study subjects (*n* = 538).

Variables	Cases	Rate(%)
Hospital level		
Level III hospitals	438	81.41
Level II hospitals	100	18.59
Education attainment		
Associate degree	137	25.46
Bachelor and above	401	74.54
Professional title		
Registered nurse	121	22.49
Nurse practitioner	291	54.09
Senior nurse and above	126	23.42
Gender		
Female	489	90.89
Male	49	09.11
Employment status		
Permanent employee	233	43.31
Contract employee	305	56.69
Marital status		
Married	362	67.28
Single	176	32.72
Parental status		
With children	366	68.03
Without children	172	31.97
Years of service		
1–5	188	34.94
>5	350	65.06
Clinical preceptorship experience		
Yes	211	39.22
No	327	60.78
PALS or PBLS		
Yes	121	22.49
No	417	77.51
Management experience		
Yes	211	39.22
No	327	60.78
Publications in the last five years		
Yes	145	26.95
No	393	73.05

### Current status of the core competencies of paediatric emergency nurses

The scores of each dimension revealed that the mean score of the professional attitude dimension was the highest, at 4.51 ± 0.62, and the mean score of the professional development competence dimension was the lowest, at 3.70 ± 0.81, as detailed in Table 2.

**Table 2:** Scoring results of each dimension of the “Self evaluation questionnaire on core competence of pediatric emergency nurses”.

Category	Average Score ± SD
Professionalism	4.51 ± 0.62
Clinical competence	3.98 ± 0.78
Comprehensive competence	4.04 ± 0.73
Professional development competence	3.70 ± 0.81
Total	4.06 ± 0.74

### Univariate analysis of the core competencies of paediatric emergency nurses

The results of the univariate analysis revealed statistically significant differences in the overall scores of paediatric emergency nurses’ core competencies across hospital level, years of experience, participation in PALS or PBLS training courses, and emergency management experience, as shown in Table 3.

**Table 3:** The relationship between general information and core competency self-assessment questionnaire entries.

Variables	Total	Professionalism	Clinical competence	Comprehensive competence	Professional development competence
Hospital level					
Level III	127.31 ± 18.92	18.14 ± 2.43	57.13 ± 9.91	40.85 ± 6.97	11.19 ± 2.32
Level II	116.09 ± 24.61	17.54 ± 2.69	49.49 ± 13.17	38.43 ± 8.61	10.63 ± 2.89
t	4.278	2.180	5.458	2.619	1.807
P	<0.001	0.030	<0.001	0.010	0.073
Education attainment					
Associate degree	125.52 ± 21.36	18.34 ± 2.23	55.91 ± 10.97	40.11 ± 7.64	11.16 ± 2.64
Bachelor and above	125.12 ± 20.28	17.92 ± 2.57	55.64 ± 11.01	40.5 ± 7.26	11.06 ± 2.38
t	0.196	1.720	0.243	−0.534	0.416
P	0.845	0.086	0.808	0.593	0.678
Professional title					
Registered nurse	127.98 ± 21.62	18.46 ± 2.59	57.33 ± 11.27	41.12 ± 7.88	11.07 ± 2.66
Nurse ractitioner	124.20 ± 18.80	17.95 ± 2.27	55.38 ± 10.20	39.94 ± 6.76	10.93 ± 2.37
Senior nurse and above	124.94 ± 23.11	17.79 ± 2.82	54.9 ± 12.34	40.78 ± 8.10	11.46 ± 2.37
F	1.462	2.568	1.789	1.314	2.070
P	0.233	0.078	0.168	0.270	0.127
Gender					
Female	125.43 ± 20.85	17.99 ± 2.53	55.81 ± 11.08	40.51 ± 7.44	11.12 ± 2.48
Male	123.16 ± 17.22	18.37 ± 2.07	54.73 ± 10.12	39.29 ± 6.48	10.78 ± 2.03
t	0.735	−1.001	0.650	1.112	0.931
P	0.463	0.317	0.516	0.267	0.353
Marital status					
Married	125.9 ± 20.6	18.07 ± 2.37	55.89 ± 11.14	40.74 ± 7.36	11.19 ± 2.47
Single	123.84 ± 20.41	17.94 ± 2.73	55.33 ± 10.69	39.69 ± 7.33	10.88 ± 2.39
t	1.091	0.559	0.557	1.555	1.393
P	0.276	0.576	0.578	0.121	0.164
Employment status					
Permanent Employee	125.68 ± 20.22	18.15 ± 2.4	56.32 ± 10.4	40.2 ± 7.52	11 ± 2.35
Contract Employee	124.87 ± 20.81	17.93 ± 2.55	55.24 ± 11.41	40.55 ± 7.24	11.15 ± 2.52
t	0.451	0.996	1.132	−0.545	−0.673
P	0.652	0.319	0.258	0.586	0.501
Parental status					
With children	125.79 ± 20.39	18.04 ± 2.53	55.92 ± 10.84	40.63 ± 7.35	11.2 ± 2.47
Without children	124.01 ± 20.87	18 ± 2.41	55.25 ± 11.32	39.91 ± 7.36	10.84 ± 2.39
t	0.941	0.178	0.663	1.052	1.579
P	0.347	0.859	0.508	0.293	0.115
Years of service					
1–5	122.34 ± 21.39	18.00 ± 2.57	54.4 ± 11.13	39.09 ± 7.90	10.85 ± 2.54
>5	126.77 ± 19.93	18.04 ± 2.45	56.41 ± 10.87	41.11 ± 6.95	11.21 ± 2.39
t	−2.394	−0.190	−2.023	−2.946	−1.632
P	0.017	0.849	0.044	0.003	0.103
Clinical preceptorship experience					
Yes	127.34 ± 20.2	18.27 ± 2.34	57.31 ± 10.06	40.67 ± 7.72	11.09 ± 2.65
No	123.86 ± 20.67	17.87 ± 2.57	54.67 ± 11.44	40.23 ± 7.12	11.09 ± 2.31
t	−1.923	−1.817	−2.814	−0.680	0.001
P	0.055	0.070	0.005	0.497	0.999
PALS or PBLS					
Yes	129.40 ± 19.91	17.83 ± 2.99	58.89 ± 9.42	41.48 ± 7.00	11.20 ± 2.35
No	124.01 ± 20.59	18.09 ± 2.33	54.78 ± 11.25	40.09 ± 7.44	11.05 ± 2.48
t	2.553	−0.882	4.036	1.838	0.576
P	0.011	0.379	<0.001	0.067	0.565
Management experience					
Yes	128.05 ± 20.28	18.00 ± 2.65	56.46 ± 10.89	41.96 ± 6.93	11.63 ± 2.36
No	123.39 ± 20.54	18.04 ± 2.39	55.22 ± 11.04	39.39 ± 7.46	10.74 ± 2.44
t	2.581	−0.173	1.275	4.012	4.177
P	0.010	0.863	0.203	<0.001	<0.001
Publications in the last five years					
Yes	125.44 ± 20.69	17.68 ± 2.94	55.54 ± 10.86	40.76 ± 7.02	11.46 ± 2.25
No	125.14 ± 20.51	18.16 ± 2.29	55.77 ± 11.05	40.27 ± 7.48	10.95 ± 2.5
t	0.151	−1.751	−0.218	0.687	2.177
P	0.880	0.081	0.827	0.492	0.030

### Multiple regression analysis of the core competencies of paediatric emergency nurses

Variables with *P* < 0.05 in the single factor analysis were included in the multifactorial linear regression model, which included six variables: hospital level, years of work experience, experience in teaching, experience in PALS and PBLS training, experience in emergency management, and publications in the last 5 years. The variable assignment table is shown in Table 4. The hospital level influenced professional attitudes. The hospital level, more than 5 years of work experience, participation in clinical teaching, and participation in PALS or PBLS training courses were influenced clinical professional practice competence. The hospital level and emergency-related management experience influenced general competence. The most influential factor in professional development competence was emergency-related management experience. The hospital level and emergency-related management experience were the factors that influenced the responses to the self-assessment questionnaire on core competence (see Table 5).

**Table 4:** Multi factor linear regression model independent Variable assignment table.

Independent variable	Assignment status
Hospital level	Level II = 0; Level III = 1
Years of service	1–5 = 0; >5 = 1
Clinical preceptorship experience	No = 0; yes = 1
PALS or PBLS	No = 0; yes = 1
Management experience	No = 0; yes = 1
publications in the last five years	No = 0; yes = 1

**Table 5:** Results of multivariate linear regression model.

Model	Variables	*β*	SE	Standardized Regression Coefficient	t	*P*
A	Constant	17.540	0.248	—	70.718	<0.001
	Level III hospitals	0.599	0.275	0.094	2.180	0.030
B	Constant	45.043	1.506	—	29.902	<0.001
	Level III hospitals	5.961	1.217	0.211	4.896	<0.001
	Years of Service: >5	3.582	1.224	0.156	2.927	0.004
	Clinical Preceptorship Experience	4.977	1.176	0.221	4.231	<0.001
	PALS or PBLS	3.149	1.140	0.120	2.763	0.006
C	Constant	37.557	0.754	—	49.783	<0.001
	Level III hospitals	2.290	0.799	0.121	2.864	0.004
	Management Experience	2.495	0.637	0.166	3.918	<0.001
D	Constant	10.675	0.141	—	75.871	<0.001
	Management Experience	0.772	0.227	0.154	3.407	0.001
	Publications in the Last Five Years	0.249	0.249	0.045	1.000	0.318
E	Constant	114.134	2.205	—	51.767	<0.001
	Level III hospitals	10.243	2.278	0.194	4.497	<0.001
	Years of Service: >5	0.933	1.988	0.022	0.469	0.639
	PALS or PBLS	2.939	2.166	0.060	1.357	0.175
	Management Experience	3.774	1.857	0.090	2.032	0.043

A Professional Attitude model F = 4.753, *P* = 0.030; *R*^2^ = 0.0088, Adjusted *R*^2^ = 0.0069.

B Clinical Professional Practice Ability model F = 14.620, *P* < 0.001; *R*^2^ = 0.1208, Adjusted R² = 0.1125.

C comprehensive communication management ability model F = 16.099, *P* < 0.001; *R*^2^ = 0.0438, Adjusted *R*^2^ = 0.0403.

D Professional Development Ability model F = 6.601, *P* < 0.001; *R*^2^ = 0.0358, Adjusted *R*^2^ = 0.0303.

E the total score of self-assessment questionnaire model F = 8.506, *P* < 0.001; *R*^2^ = 0.06, Adjusted *R*^2^ = 0.053.

## Discussion

The results of this study show that the overall mean score of the paediatric emergency nurses’ core competence questionnaire is 4.06, which is at an average level, indicating that the overall level of the core competence of paediatric emergency nurses still needs improvement. Among the four dimensions, the score for professional attitude was the highest at 4.51, significantly higher than that of the other dimensions. This finding indicates that paediatric emergency nurses love their work, are dedicated, and enjoy helping the injured. They can implement humanized nursing care by focusing on the families of the affected children. Among the four dimensions, the scores for clinical professional practice ability and comprehensive ability are at the middle level, 3.98 and 4.04, respectively. This result indicates that paediatric emergency nurses have a level of clinical professional practice ability and comprehensive ability, which can meet the needs of daily clinical work to a certain extent. However, there is still room for improvement and upward movement. Among the four dimensions, professional development ability has the lowest score of 3.70, which is the most noteworthy and concerning aspect. The low score may be related to the following reasons: (1) Organizational culture constraints: scientific research indicators are often given little weight in performance evaluation systems, with weak incentive mechanisms, and the proportion of nurses with master's degrees in emergency departments is relatively low, resulting in overall weaker research capabilities; (2) Insufficient training resources: emergency department nurses place greater emphasis on the cultivation of emergency techniques and skills in various training programs, with fewer opportunities to participate in research training; (3) Differences in professional cognition: most pediatric emergency nurses still believe that the main task of nurses is direct patient care, while they do not pay enough attention to other role functions of nurses, such as being researchers and practice improvers, health promoters and educators, managers and decision-makers; (4) High-intensity work and high-pressure environment: the rapidly changing conditions of emergency pediatric patients and the high demands of patients’ families pose significant challenges to pediatric emergency nurses, leading to encroachment on their learning time and causing cognitive fatigue, thereby affecting their learning time and learning ability. Within the clinical professional practice competencies, pre-hospital emergency care ability also ranked among the bottom three, which may be related to variations in emergency department configurations across different children's hospitals. The Guidelines for the Construction and Management of Emergency Departments suggest that the emergency department is effectively connected with prehospital emergency care, the emergency department is included in the prehospital emergency care network, and prehospital emergency care is managed according to the relevant provisions; these guidelines do not strictly define the prehospital emergency care setup. However, the work of the emergency department and the prehospital emergency department is closely related, and the methods for effective emergency information transmission, handover of paediatric emergency patients, exchange and rotation of nurses and other management work have to be further standardized and unified (1).

The core competencies of paediatric emergency nurses are affected by a variety of factors. The results of the study revealed that the total score of the questionnaire, professional attitudes, clinical professional practice ability, and the comprehensive ability of paediatric emergency nurses in tertiary hospitals were greater than those of paediatric emergency nurses in secondary hospitals, with *P* < 0.05. In terms of professional attitudes, tertiary hospitals pay more attention to the construction of hospital culture and cultivating nurses’ dedication and sense of service. In terms of clinical professional practice, tertiary hospitals offer better teaching environments and conditions, providing nurses with better educational resources (*P* < 0.05). Some tertiary hospitals have introduced advanced high-fidelity simulation teaching systems and VR teaching equipment (2). Moreover, most tertiary hospitals are affiliated with hospitals at universities, allowing teachers to receive more training in teaching methods, teaching content, and other aspects of education. The comprehensive ability of tertiary hospitals includes critical thinking, communication and coordination, and management skills.

Additionally, tertiary hospitals may have more outpatient emergency cases involving children than secondary hospitals do; paediatric emergency nurses encounter more children with relatively critical conditions, complex health conditions, and unexpected accidents or emergencies, and the nurses are more experienced (2). In emergencies, the nurses are more experienced (3). The nurses are also more able to provide emergency treatment, communicate effectively, implement psychological care for children and their families, and effectively manage people, materials, and situations.

The results of the study revealed that the total questionnaire score, clinical professional practice ability, and comprehensive capabilities of nurses with over 5 years of experience in paediatric emergency medicine were higher than those of nurses who had worked in paediatric emergency medicine for 1–5 years (*P* < 0.05). In this study, paediatric emergency medicine nurses who had worked for 1–5 years were categorized into the low-seniority group, and those who had worked for more than 5 years were categorized into the high-seniority group. Nurses in the high-seniority group had more clinical work experience, their clinical professional practice ability steadily improved, and their practices improved (4). In terms of comprehensive ability, paediatric emergency nurses’ personal psychological quality has been refined, and they are more adept at thinking differently, better able to judge situations and resolve them appropriately, more tolerant of other people's shortcomings, and their communication ability has gradually improved (5). They are better able to manage their emotions. Therefore, paediatric emergency nursing managers should address low-seniority nurses by being more understanding and providing more training opportunities, encouragement, tolerance, support and communication to allow space for growth. This method does not pull the seedlings before they grow up and achieves twice the result with half the effort.

The results of the study revealed that the clinical professional practice ability of paediatric emergency nurses who participated in clinical teaching work was greater than that of those who did not participate, *P* < 0.05. Clinical teaching work refers to the process of socialization and professional training of nursing students by clinical teaching faculty (6). To improve the quality of teaching, clinical teaching faculty will pay more attention to their professional knowledge and skills to improve their clinical professional practice ability. Therefore, to improve the clinical professional practice capabilities of paediatric emergency nurses under the premise of meeting the admission standards of clinical teaching qualifications, paediatric emergency nursing managers should reasonably allocate clinical teaching tasks, avoid rigid adherence to clinical teaching qualifications, and conduct regular evaluations of clinical teaching qualifications (6).

The results of the study revealed that nurses who participated in PALS or PBLS training courses had greater clinical practice competence than those who did not participate (*P* < 0.05). PALS and PBLS training courses are paediatric life support training courses jointly founded by the American Heart Association and the American Academy of Paediatrics (7). PALS and PBLS training courses have been widely used in China since they were introduced in 2000 (8–10). The clinical professional practice abilities of the participating healthcare personnel improved significantly, aligning with the results of this study. This finding suggests that PALS and PBLS training courses are more suitable for paediatric emergency nurses and that they improve their clinical professional practice ability after they participate in training. Training methods for paediatric emergency nurses include mainly in-hospital and out-of-hospital training. In-hospital training includes training at the hospital level and department level. In-hospital training is affected by the format and teachers’ qualifications, and the effect is not good. Out-of-hospital training mainly includes academic conferences and training courses. At present, there are no paediatric emergency nurse training courses in China, and most of the contents of the emergency nurse training courses focus on adults, with less paediatric-related content (11). The contents of paediatric nurse training courses cover various paediatric subspecialties and have less emergency-related content (12). Although the PALS and PBLS training courses can improve the clinical and professional practice ability of paediatric emergency nurses, the training time is short (PALS two and a half days, PBLS two and a half days, PALS two and a half days). The PALS training course lasts two and a half days, the PBLS training course lasts half a day, and the training object is the integration of medicine and nursing, which lacks comprehensiveness and relevance. There is a lack of systematic, comprehensive and specialty-specific training forms and content for paediatric emergency nurses. With the development of nursing specialization, there is a need to cultivate paediatric emergency nurses in the future and carry out relevant continuing education programs so as to improve the core competence of paediatric emergency nurses comprehensively.

The results of the study revealed that the total questionnaire score, comprehensive ability and professional development ability of paediatric emergency nurses with emergency-related management experience were greater than those without relevant experience (*P* < 0.05). Paediatric emergency nursing managers are clinical managers at the grassroots level, and they assume the duties of directing, organizing, supervising, and managing clinical frontline nursing work (13), which requires strong competence. In view of the duties of nursing managers, comprehensive ability is also practised in the work and constantly improved. In addition, nursing managers also undertake teaching, training, scientific research, team building and other responsibilities (14). To better complete the relevant work, nursing managers pay more attention to the cultivation of their own professional development capabilities, and their personal development goals are clear. The selection conditions for nursing management positions in some hospitals also tend to consider scientific research achievements and teaching achievements, prompting nursing staff to emphasize the cultivation of scientific research and teaching ability.

The results of the study revealed that paediatric emergency nurses who published articles in journals in the last 5 years had greater professional development ability than those who had not published articles (*P* < 0.05). Nursing research is very important work, is the basis for the development of nursing, and plays an important role in promoting the development of the nursing discipline (15). The results of foreign researchers Martinez (16) showed that nurses’ participation in scientific research activities can improve nurses’ scientific research ability, which is consistent with the results of this study; paediatric emergency nurses who have published in journals in the last 5 years demonstrate greater scientific research abilities than do nurses who have not published any papers. Research is a short board and weak point of nursing, and the reasons hindering the development of nursing research may be related to the late start of nursing research, fewer highly educated nurses, busy paediatric emergency work, and weak awareness of research (17,18). The short board is the status quo, but it must not be an excuse; Pediatric emergency nursing managers should prioritize research and foster a research-oriented mindset among nursing staff from the outset. It is proposed that research-related content be systematically integrated into routine nursing activities, including meetings, training sessions, and clinical rounds. A dedicated research protection period should be established within the nursing training programs to safeguard time for research activities. Additionally, a structured approach to encouraging daily literature review among nurses should be implemented, with a defined frequency target and an incentive mechanism linking participation frequency to performance evaluation and rewards. Specialized training in research methodology and knowledge should be provided, with opportunities for key nursing personnel to attend advanced training programs. Nurses should also be encouraged to pursue higher academic qualifications to enhance their research capabilities. Leveraging the rich data available in pediatric emergency care to conduct robust research can significantly advance the field of pediatric emergency nursing.

### Limitations

Although a combination of convenience sampling and stratified strategy was employed, potential sampling biases remain in this study. Firstly, the overrepresentation of tertiary hospitals (81.4%) compared to secondary hospitals might lead to an overestimation of overall core competency scores. Secondly, the relatively limited sample size from western remote regions (12.1%) may affect the generalizability of findings to resource-limited settings.

## Conclusions

This multicenter cross-sectional study systematically evaluated the core competencies of pediatric emergency nurses in China and their key determinants. The findings revealed that pediatric emergency nurses demonstrated moderate overall core competency levels (mean score 4.06 ± 0.74), with professional attitude representing the strongest dimension (4.51 ± 0.62) while professional development capability emerged as the most significant area requiring improvement (3.70 ± 0.81). Multivariate analysis identified hospital tier, clinical experience exceeding five years, participation in clinical teaching, completion of PALS/PBLS training, and emergency management experience as statistically significant predictors of core competency levels (all *p* < 0.05). Based on these empirical findings, we recommend nursing administrators implement tiered training programs tailored to different hospital levels, with particular emphasis on enhancing research capabilities through integrating scholarly activities into routine clinical practice, allocating protected time for research endeavors, establishing performance-based incentives for academic productivity, providing advanced methodology training for key personnel, and supporting continuing education initiatives. Concurrently, expanding clinical preceptorship opportunities for junior nurses and implementing systematic management role rotations would further develop clinical and leadership competencies. These evidence-based recommendations, grounded in our study findings, provide a comprehensive framework for improving pediatric emergency nursing education and practice, with significant potential to enhance the quality of emergency care for pediatric populations across China. The study contributes valuable empirical data to inform nursing professional development strategies while acknowledging limitations including its cross-sectional design and sampling characteristics that may affect generalizability, suggesting the need for future longitudinal investigations to further validate these findings.

## Data Availability

The raw data supporting the conclusions of this article will be made available by the authors, without undue reservation.
